# LIBS-Based Chemometric and Machine-Learning Approach for Detecting Adulteration in Açaí Pulp

**DOI:** 10.1021/acsomega.6c06030

**Published:** 2026-08-20

**Authors:** Marcus V. S. Farias, Gustavo Nicolodelli, Jorge D. M. Kondo, Jader S. Cabral

**Affiliations:** † 28119 UFU - Universidade Federal de Uberlândia, Programa de Pós-Graduação em Física, Ubelândia, Minas Gerais 38408-100, Brazil; ‡ 28117 UFSC − Universidade Federal de Santa Catarina, Programa de Pós-Graduação em Física, Florianópolis, Santa Catarina 88035-972, Brazil

## Abstract

Food adulteration remains a major challenge in the commercialization of high-value agricultural products, creating a demand for rapid and reliable analytical screening methods. In this study, Laser-Induced Breakdown Spectroscopy (LIBS) combined with chemometric and machine-learning approaches was investigated for the detection of adulterants in commercial açaí (*Euterpe oleracea* Mart.) pulp. Controlled adulteration was performed using wheat flour, corn starch, and a commercial stabilizer at mass fractions of 2%, 4%, 6%, and 10% (w/w). The acquired LIBS spectra were preprocessed through correlation-based outlier removal followed by Standard Normal Variate (SNV) normalization. Principal Component Analysis (PCA) was subsequently applied for dimensionality reduction, with approximately 25 principal components required to explain nearly 95% of the total spectral variance. Supervised classification was then performed using a Random Forest (RF) algorithm applied to the PCA-reduced data set in order to discriminate between pure and adulterated samples. The classification performance was found to depend strongly on the adulteration level, yielding accuracies of 75%, 69%, 44%, and 81.2% for the 2%, 4%, 6%, and 10% concentrations, respectively. The best performance was achieved for the 10% adulteration level, where all pure açaí samples were correctly identified. The lower performance observed at intermediate concentrations indicates increased spectral overlap between pure and adulterated samples under these conditions. Overall, the results demonstrate that LIBS combined with chemometric and machine-learning techniques constitutes a promising strategy for rapid elemental fingerprinting and screening of adulteration in açaí pulp. These findings reinforce the potential of spectroscopic–chemometric approaches as practical tools for food authentication and quality control in high-value agricultural products.

## Graphical abstract

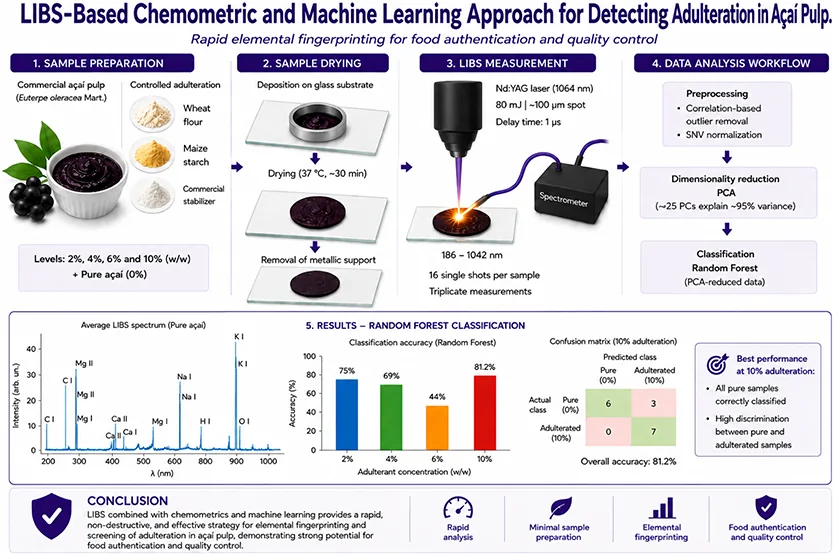

## 1 Introduction

Açaí (*Euterpe oleracea* Mart.), a fruit native to the Amazon biome, plays an important economic, social, and cultural role in the northern regions of Brazil, particularly in the states of Pará, Maranhão, Amapá, Acre, and Rondônia.^1–3^ Beyond its regional importance, açaí pulp has gained national and international recognition owing to its high nutritional value, including elevated concentrations of polyunsaturated fatty acids, dietary fibers, and antioxidant compounds such as anthocyanins.^4^ Since the 1990s, the expansion of managed plantations and cultivated areas has significantly increased fruit production, consolidating açaí as a high-value commercial commodity.^2^

The growing demand and commercial value of açaí pulp have also intensified fraudulent practices, particularly product adulteration.^5,6^ Such adulteration commonly involves the addition of low-cost substances, including wheat flour, corn starch, stabilizers, and artificial colorants, with the aim of increasing product volume and economic profits.^7^ These practices represent a recurring problem in high-value food markets and reinforce the need for rapid, reliable, and minimally invasive analytical methods capable of detecting compositional modifications in food products.^5^

Spectroscopic techniques have emerged as powerful tools for food authentication due to their rapid response, minimal sample preparation requirements, and compatibility with multivariate data-analysis methods.^7^ Among these approaches, fluorescence spectroscopy has been extensively employed in food-quality assessment because of its high sensitivity and nondestructive nature.^8,9^ In particular, the integration of fluorescence spectroscopy with chemometric techniques such as principal component analysis (PCA) has demonstrated strong potential for the analysis of complex biological matrices, including dairy products and beverages.^10–13^

More recently, laser-induced breakdown spectroscopy (LIBS) has attracted considerable attention as a rapid multielemental analytical technique capable of providing elemental fingerprints with minimal or no sample preparation.^14,15^ LIBS has been successfully applied to agricultural products and food-related materials, particularly when combined with chemometric and machine-learning approaches.^16–18^ Its capability for direct solid analysis, fast spectral acquisition, and compatibility with automated classification methods make LIBS especially attractive for food authentication and quality control applications.

In this context, the present study investigates the potential of LIBS combined with chemometric analysis for the detection of adulterants in açaí pulp samples.^14,16,17^ Controlled adulteration was performed using wheat flour, corn starch, and commercial stabilizers at different mass concentrations. The acquired LIBS spectra were analyzed using Principal Component Analysis (PCA), Linear Discriminant Analysis (LDA), and Random Forest (RF) classification in order to evaluate the discrimination and predictive performance of the proposed methodology. The main objective is to assess the feasibility of these spectroscopic and chemometric approaches as rapid and reliable tools for the authentication and quality control of açaí pulp.

## 2 Experimental Procedures

### 2.1 Sample Preparation

The samples prepared for the LIBS measurements employed commercial açaí pulp as the base matrix. Controlled adulteration was carried out through the addition of wheat flour, corn starch, and a commercial neutral stabilizer.

For each adulterant, mixtures were prepared at mass fractions of 2%, 4%, 6%, and 10% (w/w) in addition to a control sample consisting of pure açaí pulp. The inclusion of the 10% concentration extended the range investigated and enabled the evaluation of the analytical performance of the proposed methodology at higher adulteration levels. The composition of all prepared samples is summarized in Table 1.

**Table 1:** Composition of the Samples Used for LIBS Measurements

**Sample (label)**	**Composition**
Type 0	Pure açaí pulp
Type 1	Pure açaí pulp + wheat flour (2%, 4%, 6%, and 10%)
Type 2	Pure açaí pulp + stabilizer (2%, 4%, 6%, and 10%)
Type 3	Pure açaí pulp + maize starch (2%, 4%, 6%, and 10%)

Due to the high moisture content of the açaí pulp, a drying step was necessary prior to LIBS analysis. The liquid samples were deposited onto glass substrates using metallic supports to confine the material and prevent lateral spreading during deposition (see Figure 1a). Subsequently, the samples were dried in an oven at 37 °C for approximately 30 min. After the drying process, the metallic supports were removed, resulting in thin and homogeneous solid films adhered to the glass substrates (see Figure 1b). This preparation protocol improves surface uniformity and contributes to greater shot-to-shot reproducibility during plasma generation and spectral acquisition.

**Figure 1:**
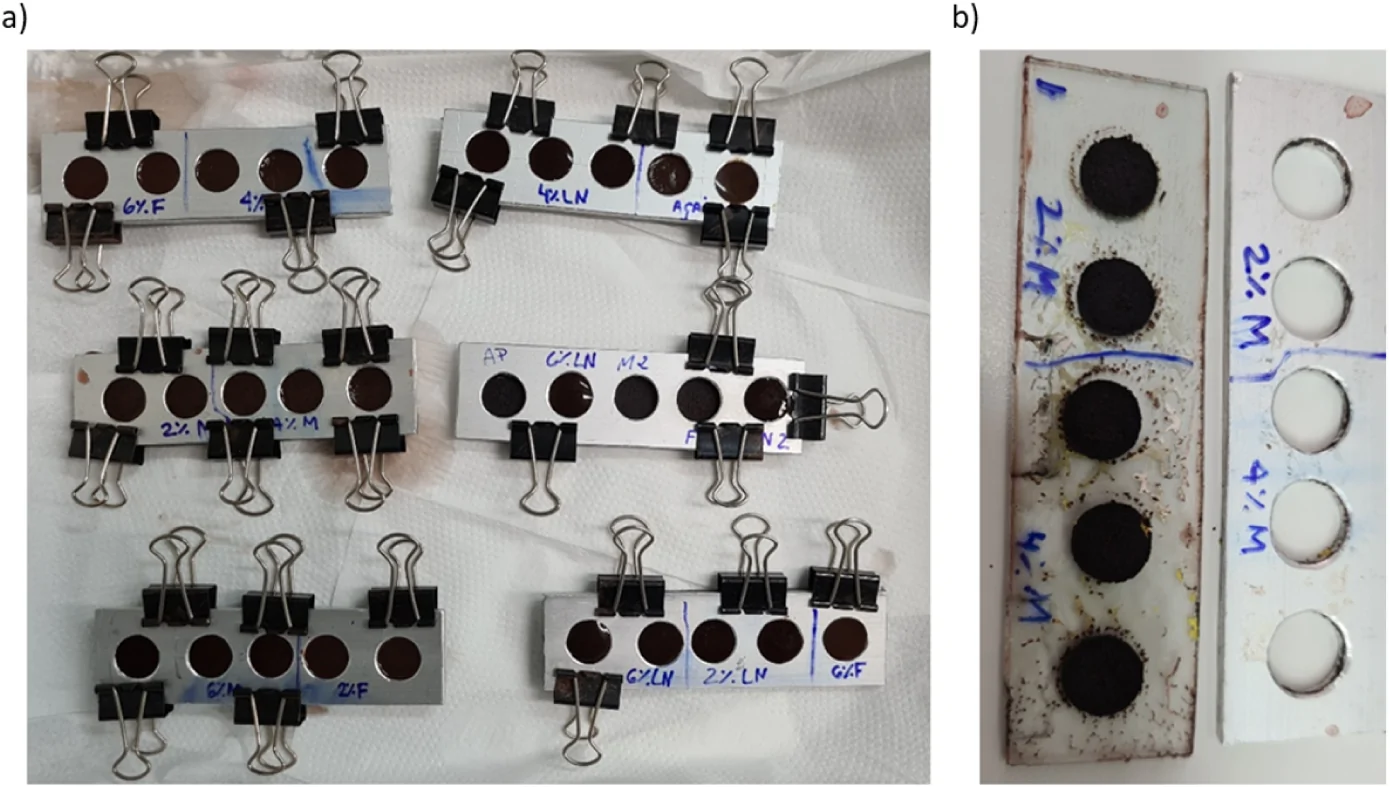
(a) Deposition of liquid samples onto glass substrates using metallic supports for confinement; (b) dried thin films obtained after removal of the metallic supports.

### 2.2 Laser-Induced Breakdown Spectroscopy (LIBS) Measurements

LIBS measurements were performed using a J200 system (Applied Spectra, USA) equipped with an Nd:YAG laser operating at a wavelength of 1064 nm (fundamental wavelength). The laser pulse energy was set to 80 mJ, with a focal spot diameter of approximately 100 μm. The delay time between plasma generation and spectral acquisition was fixed at 1 μs in order to optimize the signal-to-background ratio.^14,19^ Emission spectra were acquired over the spectral range from 186 to 1042 nm, with an approximate spectral resolution of 0.06 nm.

For each sample, 16 single-shot spectra were collected following a grid-based (4 × 4) sampling strategy. The measurement grid was positioned at the center of each dried sample to minimize any potential interference from the metallic substrate supporting the film. This approach ensured a uniform spatial distribution of the laser shots across the sample surface while avoiding edge effects. All sample conditions were independently prepared and measured in triplicate, and the resulting spectral data sets were subsequently used for statistical and chemometric analyses.

After the LIBS measurements, the spectra were subjected to a preprocessing pipeline consisting of outlier removal, followed by standard normal variate (SNV) normalization and a rolling ball algorithm to remove the background influence (baseline correction). Outliers were identified using the correlation matrix of the laser-shot spectra, and spectra presenting low mutual correlation (correlation coefficient <0.8) were excluded from the data set. Subsequently, SNV normalization was applied by using a custom Python routine. Finally, spectra corresponding to samples with the same adulterant concentration were grouped and averaged in sets of three consecutive laser shots to improve signal stability and reduce experimental variability before being used as an input for the chemometric analyses.

### 2.3 Chemometric Analyses

After preprocessing, the LIBS spectra were subjected to principal component analysis (PCA), an unsupervised multivariate statistical technique widely used for dimensionality reduction and feature extraction.^20^ This transformation enables the representation of the original data in a lower-dimensional space while preserving most of the relevant spectral information. The cumulative explained variance was subsequently calculated as a function of the number of retained principal components to determine the minimum number of PCs required to adequately represent the data set.

The selected principal components, which accounted for the majority of the total spectral variance, were then used as input features for linear discriminant analysis (LDA),^21^ aiming to maximize class discrimination while reducing data dimensionality. Applying PCA prior to LDA reduces data redundancy, minimizes the influence of noise, and helps prevent overfitting by retaining only the most informative components for the supervised classification model. LDA was employed as a supervised dimensionality reduction technique to evaluate the separability of the sample classes.

To evaluate the predictive capability of the proposed spectral analysis pipeline, the data set was formulated as a binary classification problem comprising two classes: pure açaí and adulterated açaí. Each class consisted of 23 randomly selected spectra, where each spectrum corresponded to the average of two consecutive LIBS acquisitions. The data set was divided into training and test subsets using a 2:1 ratio.

To prevent information leakage, principal component analysis (PCA) was performed exclusively on the training subset. The test spectra were subsequently projected onto the principal component subspace defined by the training data, and the resulting principal components were used as input features for the Random Forest (RF) classifier.^22^

## 3 Results and Discussion

### 3.1 Chemometric and Machine-Learning Analyses

The average LIBS spectra obtained for each adulterant concentration, together with the spectrum of pure açaí, are presented in Figure 2. Numerous electronic transitions can be observed throughout the investigated spectral range. In addition, the spectra corresponding to the different samples exhibit a high degree of similarity, making their discrimination by visual inspection alone impractical. Furthermore, no exclusive emission lines associated with the adulterated samples were identified, as all detected spectral features were also present in the pure açaí sample (Type 0). This substantial spectral overlap increases the complexity of identifying and discriminating adulterated samples, thereby reinforcing the need for chemometric and machine-learning approaches for reliable classification.

**Figure 2:**
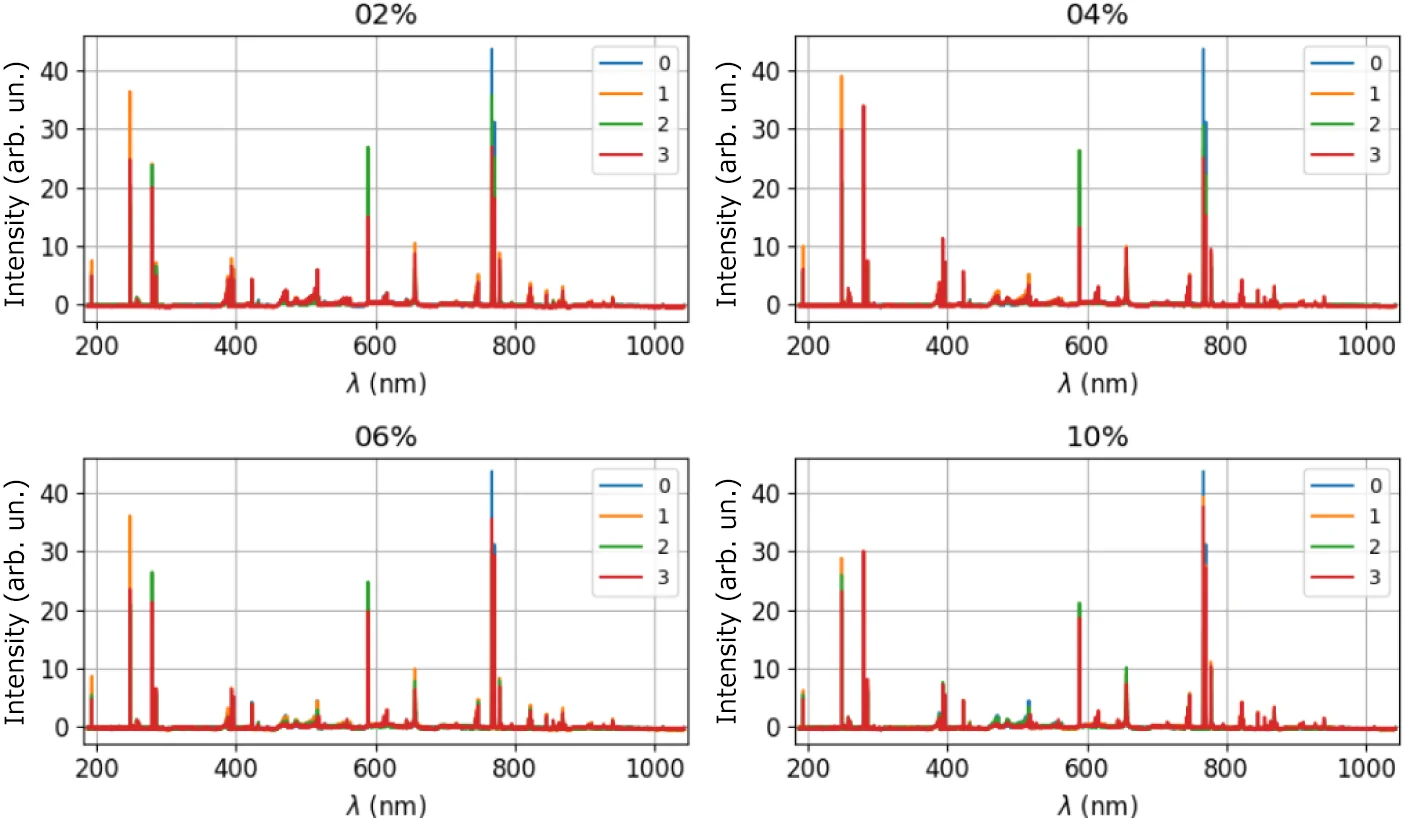
Average LIBS spectra for samples at each adulterant concentration.

As an initial step, the emission lines associated with specific chemical elements were identified. Figure 3 shows the average spectrum of an açaí pure sample with the corresponding elemental assignments for each electronic transition. Transition data were obtained from the NIST Atomic Spectra Database.^23^ The spectrum reveals the presence of C (atomic lines at 193.03 and 247.86 nm), Mg (ionic and atomic lines at 279.55, 280.27, and 285.21 nm), Ca (ionic and atomic lines at 393.37, 396.85, and 422.67 nm), K (atomic lines at 766.49 and 769.89 nm), Na (atomic lines at 588.99 and 589.59 nm), and H (atomic line at 656.27 nm), which constitute the main elemental components of the samples.

**Figure 3:**
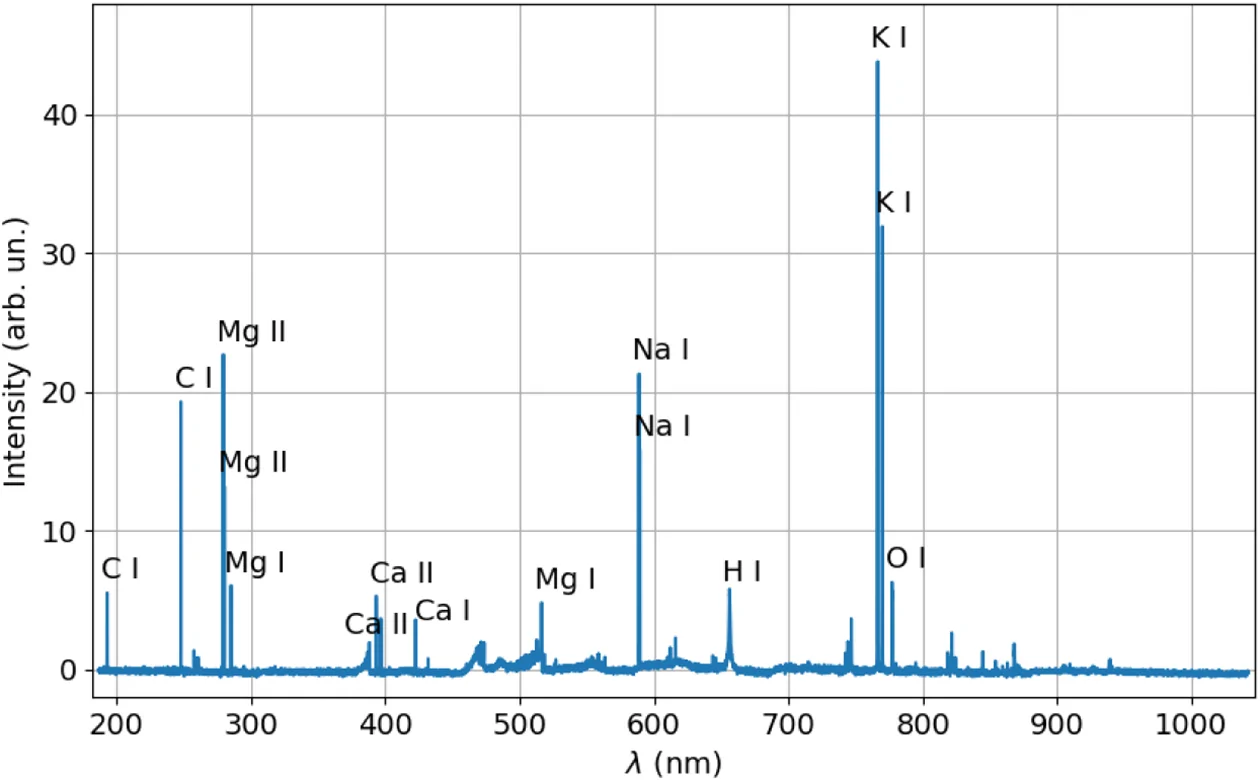
Identification of transition peaks of selected elements present in the average spectrum of the pure açaí sample.

In the following section, principal component analysis (PCA) is applied to the complete spectral data set to investigate discrimination among the different adulteration concentrations.

### 3.2 Principal Component Analysis (PCA)

Following preprocessing, principal component analysis (PCA) was applied to the spectral data set as an unsupervised dimensionality reduction technique.^20^ Figure 4a presents the score plot of the first two principal components (PC1 and PC2). At this stage, the samples are broadly distributed across the score space without forming clearly defined clusters, while the first two components account for only approximately 15% of the total variance.

**Figure 4:**
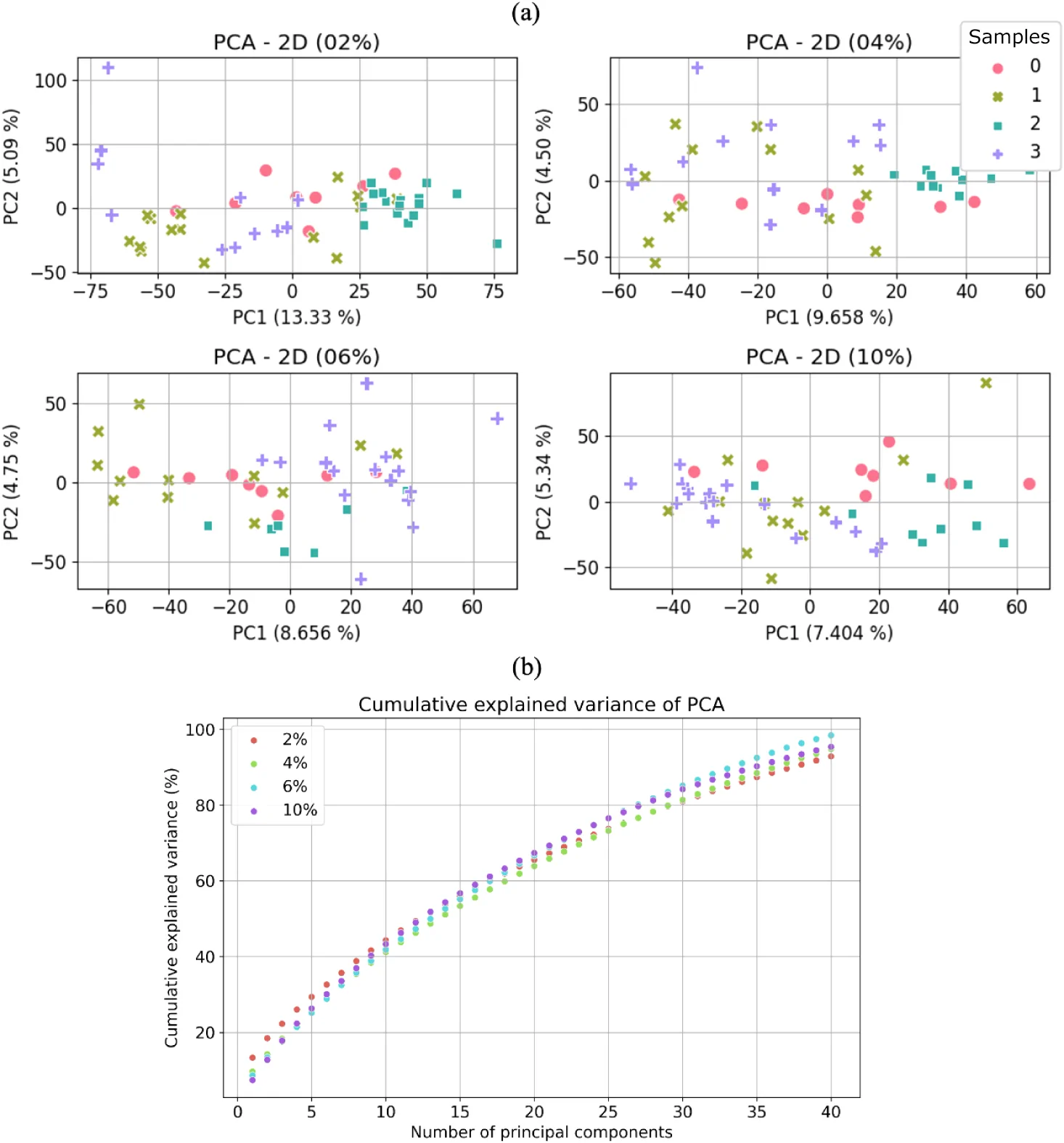
(a) PCA score map for the different adulterant concentrations; (b) cumulative explained variance as a function of the number of principal components.

Figure 4b shows the cumulative explained variance as a function of the number of principal components, indicating that nearly 40 components are required to capture more than 90% of the data set variance. On the basis of this result, the dimensionality of the data set was reduced to 40 principal components. This choice was made both to standardize the subsequent analyses and to preserve the majority of the spectral information, as summarized in Table 2.

**Table 2:** Cumulative Explained Variance of the First 40 PCA Components for Each Adulterant Concentration

Concentration of adulterant	Cumulative Explained Variance (%)
2%	92.83
4%	94.79
6%	98.43
10%	95.40

To investigate the discrimination between pure açaí and adulterated samples, the next section presents the implementation of Linear Discriminant Analysis (LDA) using the reduced feature space composed of 40 principal components.

### 3.3 Linear Discriminant Analysis (LDA)

The 40 retained principal components were subsequently used as input features for linear discriminant analysis (LDA). The projections onto the first two linear discriminants (LD1 and LD2), which together explain more than 90% of the between-class variance, are presented for each adulterant concentration level. A well-defined clustering pattern can be observed for all of the investigated concentrations, ranging from 2% to 10%.

Figure 5a shows four distinct clusters for the 2% adulterant concentration. Type 0 (pure açaí) samples are concentrated in the region characterized by negative LD1 and LD2 values, whereas Type 1 (wheat flour adulterated) samples occupy the positive-LD1 and near-zero-LD2 region. Type 3 (maize adulterated) samples are distributed close to the origin, while Type 2 (stabilizer adulterated) samples are located in the negative-LD1 and positive-LD2 quadrant. Although the Type 2 cluster exhibits greater dispersion, it remains spatially distinguishable from the other groups.

**Figure 5:**
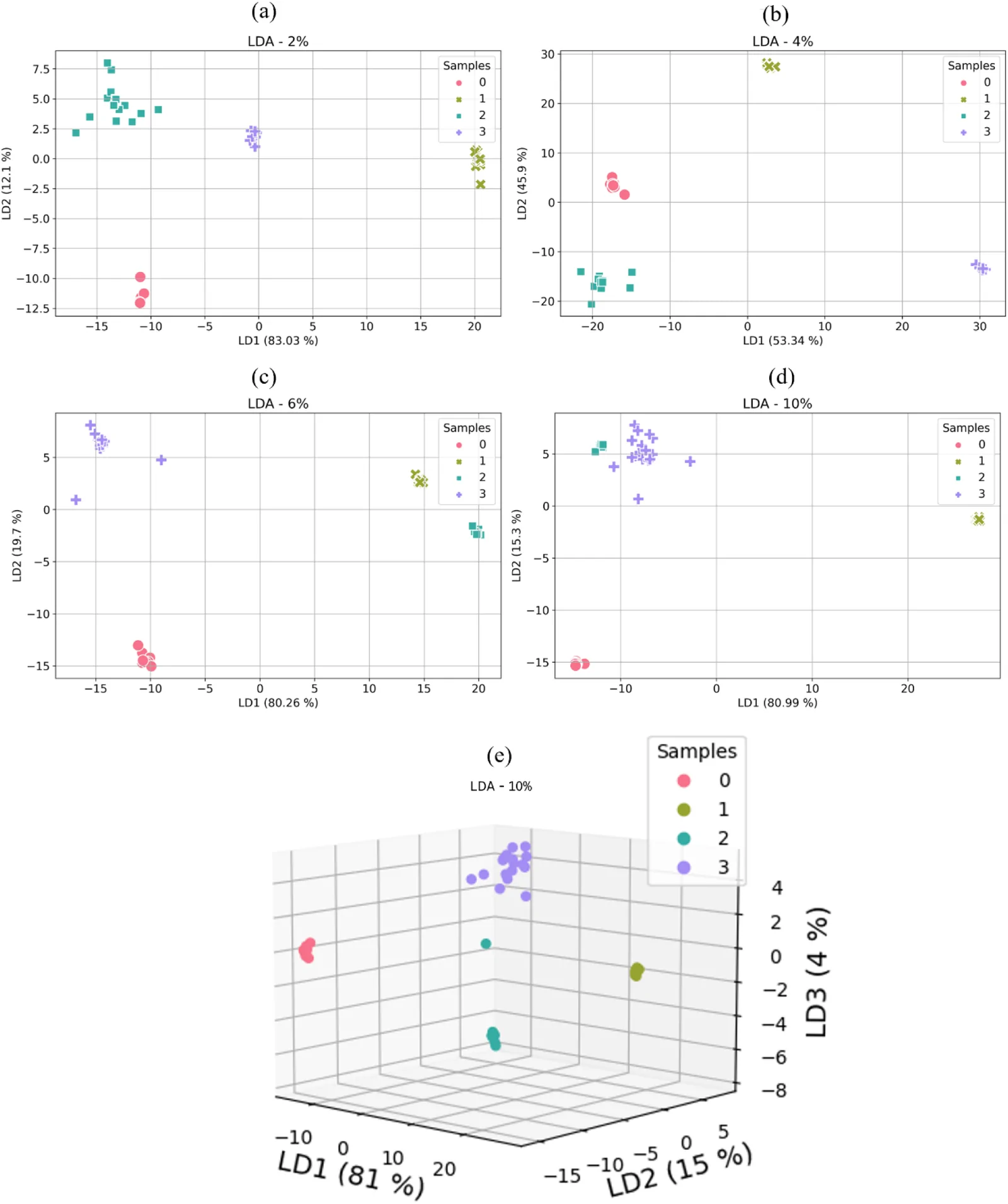
2D LDA score map for samples with (a) 2%; (b) 4%; (c) 6%, and (d) 10% adulterant concentration; (e) 3D LDA score map (LD1, LD2, LD3) for samples with 10% adulterant concentration.

At the 4% concentration level (see Figure 5b), a clear separation among the sample classes is also observed. Type 1 (wheat flour adulterated) samples are located in the positive LD1 and LD2 region, while Type 3 (maize adulterated) samples occupy the positive-LD1 and negative-LD2 region. Type 0 (pure açaí) samples are isolated in the negative-LD1 and positive-LD2 quadrant, whereas Type 2 (stabilizer adulterated) samples form a less compact but still distinct cluster with both LD1 and LD2 values negative.

For the 6% concentration (see Figure 5c), the four sample classes occupy distinct regions of the discriminant space: Type 0 (pure açaí) samples are positioned in the negative-LD1 and negative-LD2 quadrant, Type 1 (wheat flour adulterated) samples in the positive-LD1 and positive-LD2 quadrant, Type 2 (stabilizer adulterated) samples in the positive-LD1 and negative-LD2 quadrant, and Type 3 (maize adulterated) samples in the negative-LD1 and positive-LD2 quadrant. The separation among all groups is clearly defined.

At the highest adulterant concentration investigated (10%), Figure 5d reveals a pronounced separation between Type 0 (pure açaí) samples, located in the negative-LD1 and negative-LD2 region and Type 1 (wheat flour adulterated) samples, positioned in the positive-LD1 and negative-LD2 region. Type 2 (stabilizer adulterated) samples form a cluster in the upper-right portion of the score plot, in proximity to the more dispersed type 3 (maize adulterated) cluster. Although these two groups exhibit partial overlap in the two-dimensional representation, their separation becomes evident when the third discriminant component is considered, as shown in the three-dimensional score plot presented in Figure 5e.

These results demonstrate that the combination of LIBS spectroscopy with chemometric approaches—namely PCA for dimensionality reduction and LDA for supervised discrimination—constitutes a powerful strategy for the identification and classification of adulterated açaí samples.^16–18^ Clear class separation was achieved for all adulterant types and concentration levels investigated, including the lowest concentration tested (2%), highlighting the high sensitivity and discriminative capability of the proposed methodology. Although the adulterated samples did not exhibit exclusive or characteristic emission lines that would facilitate direct spectral discrimination, variations in the relative intensities of the major elemental transitions—particularly those associated with C, Mg, Ca, Na, and K—were observed among the different sample classes (see Figure 2). These subtle spectral-intensity variations constitute the primary source of discrimination captured by the PCA and LDA analyses, enabling effective differentiation between pure and adulterated açaí samples despite the strong spectral similarity among them.

In the following section, the Random Forest classification model is employed to further evaluate its capability to distinguish pure açaí samples from adulterated samples.

### 3.4 Random Forest (RF) Classification on Trained/Test Data

The predictive performance of the proposed LIBS-chemometric workflow was evaluated using an independent test set. Following the methodology described in Section 2.3, the Random Forest (RF) classifier was trained using the principal components extracted exclusively from the training subset, while the test spectra were projected onto the same PCA subspace prior to classification. The cumulative explained variance and the number of retained principal components for each adulterant concentration are summarized in Table 3. Approximately 25–26 principal components were sufficient to explain more than 95% of the spectral variance in all cases, providing a compact and informative representation of the LIBS data for the subsequent classification stage.

**Table 3:** Cumulative Explained Variance and Number of Retained PCA Components for the Training Subset at Each Adulterant Concentration

Concentration of adulterant	Cumulative Explained Variance (%)	Number of PCA components
2%	95.44	25
4%	95.70	26
6%	95.38	26
10%	96.32	25

Among the evaluated machine-learning algorithms, the Random Forest (RF) classifier exhibited the best overall performance. The principal hyperparameter considered was the number of decision-tree estimators, whose optimal value was independently determined for each adulterant concentration level. The classification results are presented as confusion matrices in Figure 6 together with the corresponding overall accuracies.

**Figure 6:**
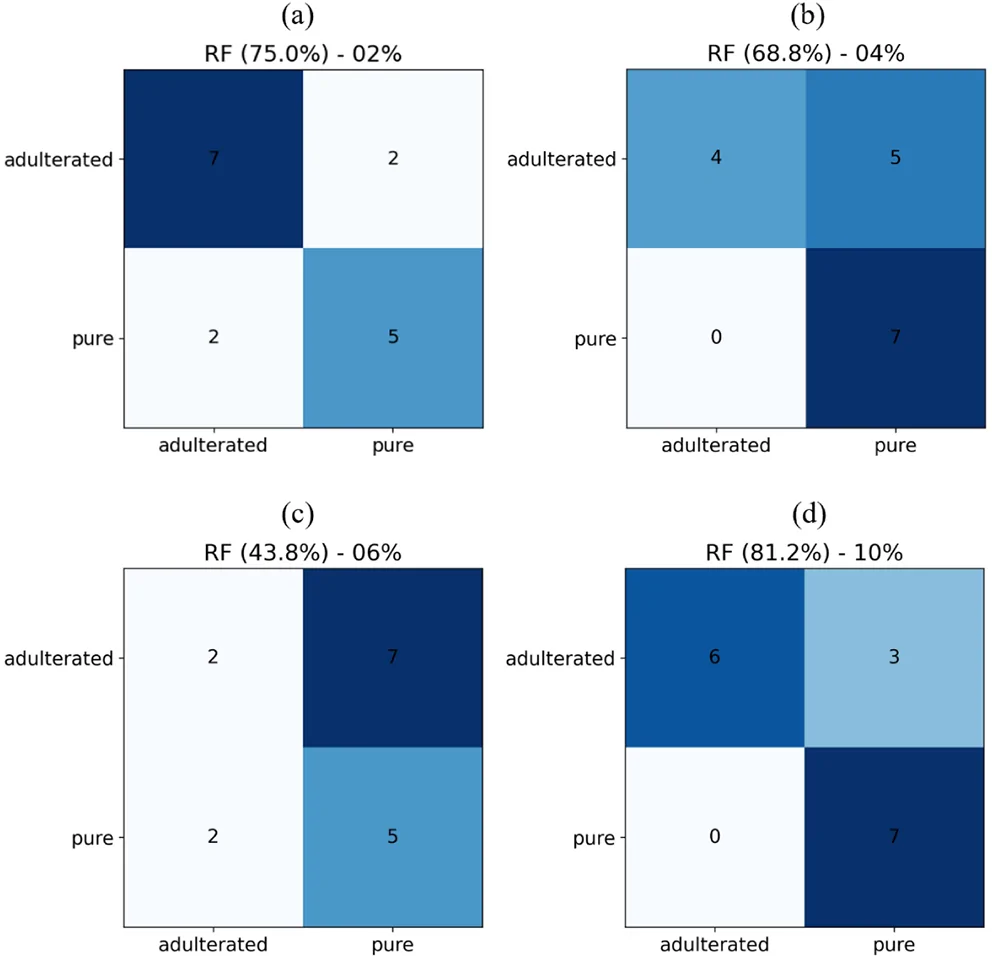
Confusion matrices obtained for the Random Forest classifier at different adulterant concentrations: (a) 2% concentration (accuracy: 75%; 8 estimators), (b) 4% concentration (accuracy: approximately 69%; 110 estimators), (c) 6% concentration (accuracy: approximately 44%; 4 estimators), and (d) 10% concentration (accuracy: 81.2%; 7 estimators).

For the 2% adulterant concentration, the optimal configuration consisted of 8 estimators, resulting in a classification accuracy of 75%, as shown in Figure 6a. At the 4% concentration level, the best performance was achieved using 110 estimators, yielding an accuracy of approximately 69%. As illustrated in Figure 6b, all pure açaí samples were correctly classified; however, more than half of the adulterated samples were incorrectly labeled as pure, indicating that the spectral changes introduced at this concentration are comparatively subtle under the binary classification framework.

The 6% concentration level represented the most challenging conditions for the RF classifier. Using 4 estimators, the model achieved an accuracy of approximately 44% (Figure 6c), which is close to the expected performance of random classification in a two-class problem. This result suggests a substantial overlap in spectral characteristics between pure and adulterated samples at an intermediate concentration.

The best classification performance was obtained for the 10% adulterant concentration. Using 7 estimators, the RF classifier achieved an accuracy of 81.2% (see Figure 6d). In this case, all pure açaí samples were correctly identified, while only three adulterated samples were misclassified as pure, demonstrating that higher adulterant concentrations generate more distinguishable spectral signatures.

An unexpected decrease in classification performance was observed for the 6% adulteration level, where the Random Forest classifier achieved substantially lower accuracy than for the other concentrations. A detailed inspection of the corresponding LIBS spectra revealed a higher level of spectral noise and greater shot-to-shot intensity variability compared with the remaining data sets. This increased variability likely reduced the discriminative information contained in the spectra, making it more difficult for the classifier to distinguish between pure and adulterated samples. Although the exact origin of this behavior cannot be conclusively established from the present study, it may be associated with experimental factors such as laser pulse-energy fluctuations, local sample heterogeneity, or slight differences in the drying process of the açaí films, all of which can influence plasma formation and spectral reproducibility. Since these factors were not systematically investigated, they should be regarded as plausible explanations rather than definitive causes.

Overall, the Random Forest classifier validated the effectiveness of the PCA-based spectral-analysis pipeline when applied to independent test data. The highest accuracy was achieved for the 10% adulteration level, where the spectral deviations relative to pure açaí are more pronounced. Future investigations may benefit from the application of multiclass classification strategies or alternative feature-extraction approaches to improve classification robustness across all concentration levels.

## 4 Conclusions

In this work, the feasibility of combining Laser-Induced Breakdown Spectroscopy (LIBS) with chemometric and machine-learning approaches for the detection of adulteration in açaí pulp was systematically investigated. Controlled adulteration using wheat flour, corn starch, and commercial stabilizers at different mass fractions enabled a comprehensive assessment of the analytical performance of the proposed methodology. The results demonstrate that LIBS provides reproducible elemental fingerprints for the investigated samples, although direct visual discrimination between pure and adulterated spectra is impractical due to their high spectral similarity. In this context, the application of multivariate analysis proved essential for extracting relevant discriminatory information from the spectral data set.

Principal Component Analysis (PCA) successfully reduced the dimensionality of the data while preserving most of the spectral variance, whereas Linear Discriminant Analysis (LDA) enabled a clear separation of the sample classes for all investigated adulteration levels, including low concentrations. These results highlight the strong discriminative capability of the proposed spectroscopic–chemometric approach.

The supervised classification performed using the Random Forest algorithm further confirmed the predictive potential of the methodology. Although intermediate adulteration levels, particularly at 6%, represented a more challenging classification scenario, the best performance was achieved for the 10% concentration level, where the spectral deviations relative to pure açaí samples become more pronounced. The unexpectedly lower classification accuracy observed at the 6% concentration is likely associated with increased spectral noise and shot-to-shot intensity variability identified in this data set, which may have reduced the discriminative information available to the classifier. Although the exact origin of this variability could not be conclusively determined, it is likely related to experimental factors, such as laser pulse-energy fluctuations or slight differences in sample preparation. Therefore, this result should be regarded as an isolated experimental limitation rather than an inherent limitation of the proposed LIBS-based chemometric and machine-learning approach.

Overall, the integration of LIBS with chemometric and machine-learning tools demonstrated significant potential as a rapid, nondestructive, and minimally invasive strategy for the screening of adulteration in açaí pulp. The proposed methodology offers important advantages, including minimal sample preparation, fast spectral acquisition, and compatibility with automated classification frameworks, making it attractive for applications in food authentication and quality control.

Future studies should focus on improving classification robustness at low adulteration levels through the incorporation of larger data sets, more controlled and homogeneous sample-preparation procedures, and alternative feature-extraction and classification strategies. Collectively, these results reinforce the potential of LIBS-based spectroscopic and chemometric methods as valuable analytical tools for the authentication of high-value agricultural products.
